# Wear Resistance of 3D Printing Resin Material Opposing Zirconia and Metal Antagonists

**DOI:** 10.3390/ma11061043

**Published:** 2018-06-20

**Authors:** Ji-Man Park, Jin-Soo Ahn, Hyun-Suk Cha, Joo-Hee Lee

**Affiliations:** 1 Department of Prosthodontics, College of Dentistry, Yonsei University, 250 Seongsanno, Seodaemun-gu, Seoul 03722, Korea; jimarn@yuhs.ac; 2 Department of Dental Biomaterials Science and Dental Research Institute, School of Dentistry, Seoul National University, 101 Daehak-ro, Jongno-gu, Seoul 03080, Korea; ahnjin@snu.ac.kr; 3 Division of Prosthodontics, Department of Dentistry, Asan Medical Center, College of Medicine, University of Ulsan, 88 Olympic-ro 43-gil, Songpa-gu, Seoul 05505, Korea; chahyunsuk@hanmail.net

**Keywords:** 3D printing, CAD/CAM, wear, resin, zirconia, metal

## Abstract

3D printing offers many advantages in dental prosthesis manufacturing. This study evaluated the wear resistance of 3D printing resin material compared with milling and conventional resin materials. Sixty substrate specimens were prepared with three types of resin materials: 3D printed resin, milled resin, and self-cured resin. The 3D printed specimens were printed at a build angle of 0° and 100 μm layer thickness by digital light processing 3D printing. Two kinds of abraders were made of zirconia and CoCr alloy. The specimens were loaded at 5 kg for 30,000 chewing cycles with vertical and horizontal movements under thermocycling condition. The 3D printed resin did not show significant difference in the maximal depth loss or the volume loss of wear compared to the milled and the self-cured resins. No significant difference was revealed depending on the abraders in the maximal depth loss or the volume loss of wear. In SEM views, the 3D printed resin showed cracks and separation of inter-layer bonds when opposing the metal abrader. The results suggest that the 3D printing using resin materials provides adequate wear resistance for dental use.

## 1. Introduction

The most recent technological movement in digital dentistry is centered on additive manufacturing called 3D printing. A 3D printing technique for the fabrication of dental prostheses offers many advantages. Because digital data acquired from the intra-oral scanner can be printed into a physical working model, the impression procedure can be skipped, which prevents patients from experiencing nausea due to swallowing the impression materials. In addition, the laboratory procedures for prosthesis fabrication can be reduced. Consequently, 3D printing helps save materials and energy, decreases the carbon footprint, and is more economical than conventional methods [1,2,3]. Subtractive manufacturing called milling shares many of the same advantages as 3D printing, i.e., the impression technique is similar and various fabrication procedures are omitted [4]. However, the milling method results in more wastage because the procedure entails cutting materials with a bur, which produces heat, noise, and an unfavorable force [5]. The use of 3D printing as a substitute for milling is thus of more interest for the manufacturing of dental prostheses.

3D printing has considerable potential for application in the dental field. 3D printing machines are now cheaper, smaller, and lighter, and can work with various materials including metals, ceramics, and polymers [6]. Recent studies have shown that dental prostheses manufactured using 3D printing method have an acceptable degree of precision compared with prostheses made using milling or conventional methods [7]. Furthermore, several studies have reported 3D printing applications in maxillofacial reconstruction [8,9,10], implant fixture construction and intervention [11], orthodontic appliances [12], metal bridges [13], guided tissue regeneration [14], tissue surfaces [15], and the metal frameworks of removable partial dentures [16]. The fabrication of dental provisional restorations is one remaining area that will benefit from these recent developments. Provisional restorations made of polymethylmethacrylate (PMMA)-based resin have been used to protect pulpal tissue from bacterial contamination, physio-mechanical and thermal irritation. Long-term use of provisional restorations is frequently necessary for a large range of occlusal reconstruction and implant treatment. In such situations, sufficient wear resistance and mechanical strength are essential for clinical use. However, conventional self-polymerizing PMMA-based resin materials showed high polymerization shrinkage, heat generation, and water sorption [17]. In addition, conventional provisional restorations are commonly fabricated using the templates filled with a mixture of self-polymerizing powder and liquid. This technique usually requires longer chair time for additional adjustments and offers lower marginal integrity. Due to the advantages of digital manufacturing, 3D printed resin materials can be an alternative to conventional resin materials for long-term dental applications.

Zirconia for full zirconia crowns and the CoCr alloy for dental restorations have been utilized widely in dental clinics. The wear occurring between provisional restorations and occluding prosthesis is a very important factor to consider in the extended temporary stage for a final restoration decision in clinical practice. For the 3D printing to be applicable in clinical practice, the wear resistance of provisional prostheses manufactured using 3D printing method should be investigated against zirconia and metal antagonists. Studies regarding the wear resistance of dental materials have been conducted on a variety of manufacturing methods and materials [18,19], but all were limited to conventional fabrication methods. It is meaningful that the wear pattern of the 3D printing material is experimented. Some comparative studies of the physical properties of materials made using the milling and the conventional method have been reported [20,21], but no comparison with the 3D printing method has been reported. This study investigated the wear patterns of three kinds of resin materials fabricated by 3D printing, milling, and conventional self-polymerizing. Measurements of volume loss and maximal depth loss of wear were performed for the wear facets of three resin materials subjected to a wear generating process through chewing simulation. The purpose of this study was to compare the wear resistance of the 3D printed resin material to the milled and the conventional self-cured resin materials opposing zirconia and metal antagonists.

## 2. Materials and Methods

### 2.1. Computer-Assisted Designing (CAD) and 3D Printing for Specimen Preparation

Three different types of resin materials were used for the wear test (C&B; NextDent, Soesterberg, Netherlands), (Vipiblock; VIPI, São Paulo, Brazil), (Jet™; Lang Dental Mfg., Wheeling, IL, USA). The mechanical properties and basic composition of the resin materials used in this study are presented in Table 1.

**Figure 1:**
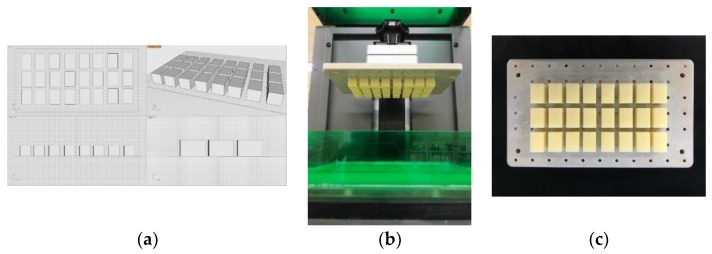
3D printed resin specimens were fabricated from a DLP 3D printer. (**a**) preprocessing before 3D printing; (**b**) printed specimens on build platform in the 3D printer; and (**c**) 3D printed specimens after primary wash.

The substrate specimens were rectangular parallelepipeds measuring 15 × 10 × 10 mm (length × width × height) in size and were designed by a universal CAD software (Rhinoceros^®^; version 5, Robert McNeel & Associates, Seattle, WA, USA). The specimens of the 3D printed resin were manufactured using a digital light processing (DLP) 3D printer (D1-150; Veltz3D, Incheon, Korea). The laser was controlled by a digital micro mirror and the entire layer of liquid resin was polymerized at once. The specimens were printed with a build angle of 0° where the side to be tested was parallel to the build platform (Figure 1). The *z*-axis layer thickness was set to 100 μm. The accuracy of the printed specimens with various thickness showed the highest values at 0° orientation, and the error was significantly low. In addition, peak stress was higher in prints with a layer thickness of 100 μm [22]. After the 3D printing process, the blocks were detached from the platform and washed with 100% isopropyl alcohol to remove excessive resin monomers. In the final stage, the specimens underwent a postcuring processing for 120 min using a postcuring machine (Denstar-300; Denstar Co., Daegu, Korea).

For the milled resin, a disc-type block was machined by a dry milling machine (DWX-51D; Roland DGA Corp., Irvine, CA, USA). The tool path of the specimen design was calculated by computer-assisted manufacturing (CAM) software (hyperDENT^®^ version 7.4, FOLLOW-ME! Technology GmbH, Munich, Germany). The resin disc was machined following the tool path, and the machined blocks were sintered. In the self-cured resin, a silicon mold was prepared and filled with resin mixed at a powder: liquid ratio of 100:52, according to the manufacturer’s recommendation. Then, the mixture was placed in the mold, covered with a glass slide, and cured in a pot containing water at a pressure of 0.21 MPa. It was obtained with 20 specimens for each of the material. Before the wear test, the specimens were dried at a temperature of 37 °C for one day. Then, the specimens were ground and polished with silicon carbide paper of grain sizes 600 and 1200 grit on a rotary machine with water cooling.

**Figure 2:**
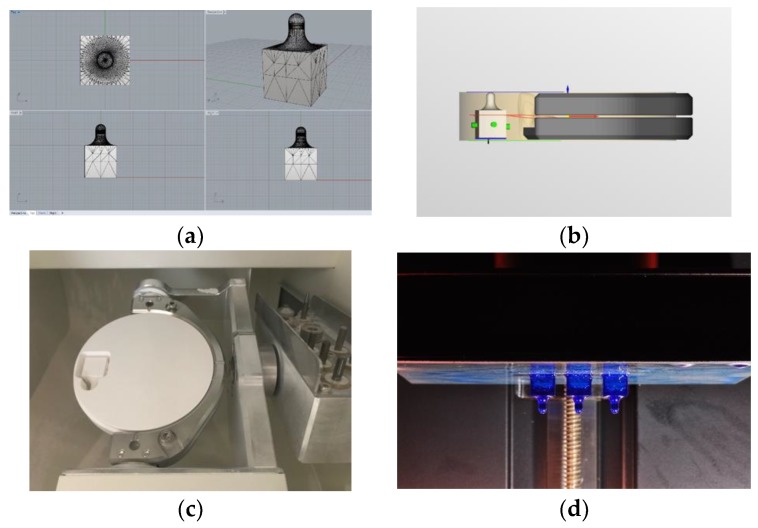
The zirconia abraders were fabricated by milling, and the metal abrader was 3D printed, invested, and cast into a CoCr alloy. (**a**) computer assisted design for the abraders; (**b**) calculation and arrangement on CAM software (hyperDENT^®^ version 7.4, FOLLOW-ME! Technology GmbH, Munich, Germany) for the zirconia abrader; (**c**) the zirconia abrader inside a block after milling; (**d**) sacrifice patterns made of castable 3D printing resin for the metal abraders.

The abrader, which was mounted on a chewing simulator applying abrasive force to the specimen, was made of zirconia and CoCr alloy. It was designed to have a hemisphere with a radius of 1.5 mm according to the cuspal radio reported [23], connected to a 10 mm cube via a 5 mm-long neck (Figure 2). In the wear tests, the mesio-palatal cusp of the upper molar was used frequently for size. As the sharpness of the antagonist is greater, the wear rate increases. The zirconia abrader was fabricated by a dry milling machine (DWX-51D; Roland DGA Corp., Irvine, CA, USA) from a disc-shaped tetragonal zirconia polycrystal-based block (ZirPremium UT+; Acucera Inc., Pochon, Korea; Vickers hardness number of 1200) and sintered. The metal abrader was 3D printed by a SLA-type machine (Form2, Formlabs Inc., Somerville, MA, USA) using castable material (Castable Resin; FormLabs Inc., Somerville, MA, USA) and cast into a CoCr alloy (Wirebond 280; Bego GmbH, Bremen, Germany; Co, 60.2%; Cr, 25.0%; Mo, 4.8%; Vickers hardness number of 280) by a lost wax technique. The metal abrader surface to be tested was polished in one direction with a 1200-grit brown rubber point (Brownie^®^ Polisher PC2, SHOFU, Kyoto, Japan). Polishing the surfaces of the zirconia abraders was performed using a polishing kit (Soft Diamonds Grinding and Buffing Wheels; Asami Tanaka Dental, Friedrichsdorf, Germany). The abraders were polished with the full series of polishing discs rotating at approximately 10,000 rpm in a slow speed handpiece [18].

### 2.2. Wear Testing and Quantitative/Qualitative Analysis of Wear

A chewing simulator (CS-4.8, SD; Mechatronik, Feldkirchen-Westerham, Germany), which can test 8 antagonists and abrader pairs simultaneously, was used in this study. Each chamber consisted of an upper sample holder on which the abrader could be fixed with a screw, and a lower plastic sample holder on which the substrate specimen was built. The parameters used in this study are presented in Table 2. The chewing cycle of the abrader was set to have 5 mm vertical descending movement, 2 mm horizontal movement, ascend, and recover to its original position. The vertical load was maintained at 5 kg during the scraping motion, equivalent to the masticating force of 49 N [24]. It was conducted under a thermocycling condition of 5–55 °C by a heat/cool system with a programmable logic. Each specimen was abraded for 30,000 cycles, which is equivalent to one and a half months of chewing from a clinical perspective

The abraded specimens were steam cleaned and air-dried to remove any specks of dirt prior to scanning. To obtain surface data, the specimens were scanned in a 3-axis blue LED light scanner (Identica Hybrid; Medit, Seoul, Korea) with an accuracy of 7 μm (ISO 12836). For the quantification of wear, loss of volume and maximal depth can be measured directly on specimens using a 3D scanning device, an optical sensor, or a profilometry device. There was a very good match between the three kinds of measuring methods to quantify, and the ranking has not been significantly affected by the quantitative methods [25]. Due to its simplicity and speed, the scanning was adopted in this study. The acquired image was imported on the universal reverse engineering software (Rapidform 2004; version, Geomagic Inc., Cary, NC, USA). The worn part of the specimen was cut out, and this shell was inverted with the “reverse normal” command. It was then precisely aligned on top of the rectangular parallelepiped measuring 6 × 5 × 0.5 mm (width × length × height) on the universal CAD S/W (Rhinoceros^®^ version 5, Robert McNeel & Associates, Seattle, WA, USA) and combined into one solid to enable volume measurement. The amount of abrasion (volume loss) by the chewing simulator was calculated by subtracting the volume of the rectangular solid meshbox, which was 15 mm^3^, from the total volume of the produced solid. The wear depth (maximal depth loss) was calculated by subtracting the height of the meshbox, which was 0.5 mm, from the total height. The qualitative wear analysis was performed on gold-sputtered replicas of the specimens with field emission scanning electron microscopy (FESEM) (Hitachi S-4700, Hitachi High-Technologies Group, Schaumburg, IL, USA) at various magnifications at the end of the wear test.

### 2.3. Statistical Analysis

Statistical analysis of the values from the materials was carried out using a statistics software (SPSS, IBM Corp., New York, NY, USA). Tests of normality and equality of variances were applied. The nonparametric Kruskal–Wallis and Mann–Whitney tests were used to analyze the data at a significance level of 5%.

## 3. Results

**Figure 3:**
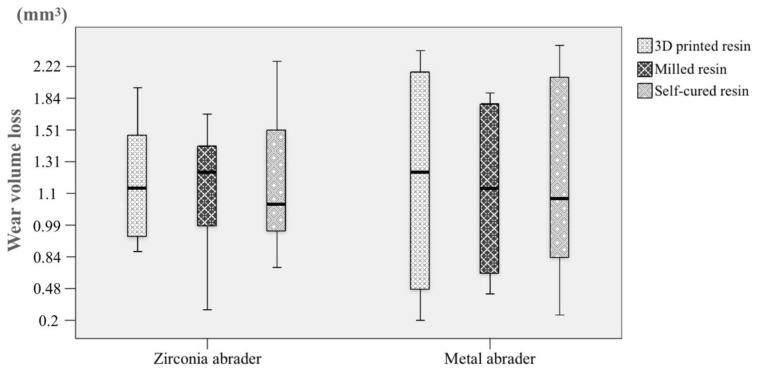
The wear volume loss of the materials against the zirconia and the metal abraders. The length of the box represents the interquartile ranges (IQRs) and the horizontal black line in the box stands for the median. The vertical lines extend to the maximum and minimum values.

**Figure 4:**
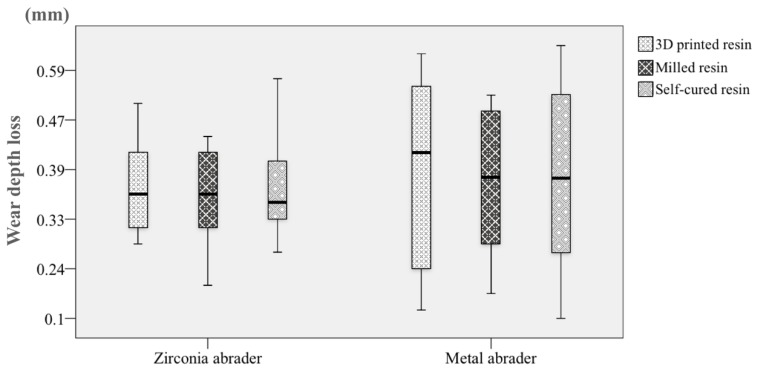
The maximal wear depth loss of the materials against the zirconia and the metal abraders. The length of the box represents the IQRs and the horizontal black line in the box stands for the median. The vertical lines extend to the maximum and minimum values.

The volume loss and the maximal depth loss of wear of the substrate specimens after the chewing cycles are presented in Figure 3 and Figure 4. The medians and interquartile ranges (IQRs) of the volume loss (mm^3^) against the zirconia abrader and the metal abrader, respectively, was 1.11 (IQR, 0.96–1.50), and 1.22 (0.47–2.20) for the 3D printed resin, 1.20 (0.90–1.42) and 1.11 (0.63–1.81) for the milled resin, and 1.06 (0.93–1.63), and 1.06 (0.73–2.30) for the self-cured resin (Figure 3). There was not any significant difference in the volume loss among the resin materials (*p* = 0.957).

The medians and IQRs of the maximal depth loss (mm) against the zirconia abrader and the metal abrader, respectively, was 0.36 (IQR, 0.32–0.43) and 0.42 (0.22–0.56) for the 3D printed resin, 0.35 (0.30–0.41) and 0.38 (0.28–0.51) for the milled resin, and 0.35 (0.32–0.41) and 0.38 (0.25–0.57) for the self-cured resin (Figure 4). Any significant difference in the maximal depth loss was not observed among the resin materials (*p* = 0.973). Furthermore, no significant difference was observed in the maximal depth loss of wear (*p* = 0.433) or volume loss of wear (*p* = 0.941) between the abraders.

**Figure 5:**
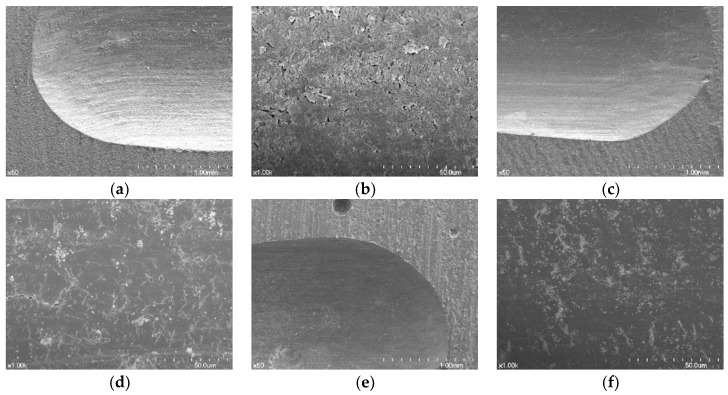
SEM images of the worn surfaces of the materials against the zirconia abrader. (**a**) 3D printed resin (original magnification ×50); (**b**) 3D printed resin (original magnification ×1000); (**c**) milled resin (original magnification ×50); (**d**) milled resin (original magnification ×1000); (**e**) self-cured resin (original magnification ×50); (**f**) self-cured resin (original magnification ×1000).

**Figure 6:**
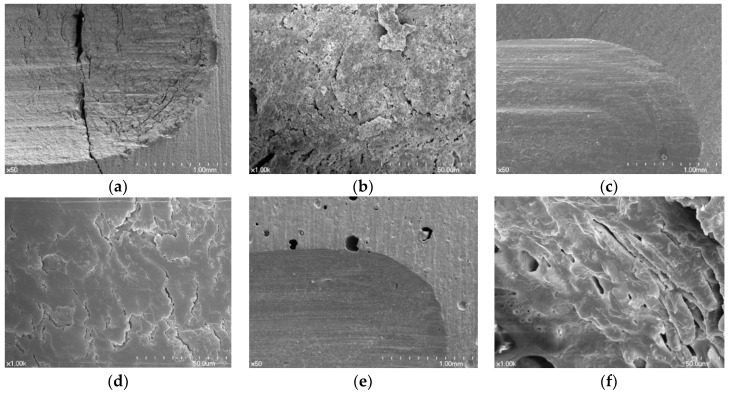
SEM images of the worn surfaces of the materials against the metal abrader. (**a**) 3D printed resin (original magnification ×50); (**b**) 3D printed resin (original magnification ×1000); (**c**) milled resin (original magnification ×50); (**d**) milled resin (original magnification ×1000); (**e**) self-cured resin (original magnification ×50); (**f**) self-cured resin (original magnification ×1000).

From the SEM images after the wear tests, the wear facets of the three resin materials showed compressed and crushed features (Figure 5 and Figure 6). Some wear pattern differences appeared among the resin materials.

In some of the 3D printed resin specimens, cracks were observed when the metal abrader was applied, and the bond between the layers was observed to be detached (Figure 6a). In the ×1000 image, fine flaws like cracks were observed and some resin particles that did not fall off remained on the layer (Figure 6b). The surfaces of the wear areas of the three materials in contact with the zirconia abrader appeared relatively smooth. The milled resin specimens were dented and showed homogenous images (Figure 5c and Figure 6c). In self-cured resin specimens, small pores were observed induced by air bubbles despite curing under pressure (Figure 5e and Figure 6e).

## 4. Discussion

As the results of this study, the 3D printing resin material showed clinically comparable wear resistance. The amount of wear of the 3D printed resin was similar to the wear amount of the milled and the conventional self-cured resins. The values of volume loss and maximal depth loss of wear showed similar patterns to each other. In the SEM images, some pores were observed in the self-cured resin specimens, and, from a clinical standpoint, these pores can have the potential to cause a defect or fracture with longer use. The wear facets showed various features: compressed and/or crushed, rough and/or smooth, and ingredients compacted to be clump together, as revealed by the SEM images of the resin specimens. The basic component of these three resin materials is the same, but differences in wear patterns were found between the materials according to the abraders. This suggests that the properties of PMMA-based resin materials may vary according to the fabrication methods. In the 3D printed resin specimens when the metal abrader was applied, cracks and fine flaws were observed. Separation of the inter-layer bonds occurred, after which the resin residues still remained attached to the lower resin layer. This seems to be due to the difference in manufacturing methods. In the 3D printing manufacturing method, layer printing is performed and bonding between layers occurs. From a mechanical point of view, inter-layer bonding is generally weaker than intra-layer bonding in 3D printed materials. The reason for the difference by the abraders seems to be due to the difference in the nature of the abrader materials. The surface roughness of the abraders affected only the beginning, and, after a certain period of contact, wear was influenced by the properties of the material itself. The zirconia particles are smoother than the non-precious metal, resulting in a difference in the results of this study.

Test parameters to investigate the wear resistance of the materials differ widely from one study to another. Most in vitro wear tests allowed ranking of materials and comparative assessment under standardized conditions, but there has been limited correlation with clinical results. Therefore, examination parameters similar to the clinical conditions are desirable. The 49 N chewing force represents the average chewing force, which has been popularly adopted for in vitro simulations of oral conditions. A horizontal movement was included to simulate oral chewing with water as the liquid medium. It was reported that cracks could be formed because the load on the sliding element caused a stress 10 times greater than the static load [26]. In this study, 2 mm horizontal movement was applied with the water chambers drained for changes between 5 °C and 55 °C. Thermal cycling has been used as a method of artificial aging to produce a wear increasing effect. Water supplying and thermocycling caused additional aging, and removed debris from the specimen surfaces keeping specimens wet during the wear test. In addition, 0.4 to 75 N ranges of force and 10,000 to 1,200,000 cycles were adopted for most wear tests. A cycle of 240,000 to 250,000 loadings in the chewing simulator is similar to that of about one year in clinical situations [27]. Thus, 30,000 cycles of load are comparable to approximately one and a half months of chewing, from a clinical perspective. In the dental field, provisional restorations are usually used for less than one and a half months. Furthermore, it was found that about 40% of the final wear occurred during the first 10,000 cycles in resin materials [28]. Most in vitro wear tests represented a progressive step where wear increased rapidly in the initial stage and then the curve became flat. The 3D printed resin material showed enough wear resistance for dental provisional restorations.

Studies on 3D printed materials have been focused on strength and accuracy. In a previous study, the elastic modulus of 3D printed resin material (C&B; NextDent, Soesterberg, The Netherlands) was found to be similar to that of conventional resin material (Jet™; Lang Dental Mfg., Wheeling, IL, USA), and the peak stress of the 3D printed resin material was significantly greater than that of the conventional resin material [22]. In our study, the wear resistance of the material fabricated by 3D printing was evaluated, and the results showed that the 3D printed resin material could yield stable clinical outcomes comparable to those of the milled or the self-cured resins. Clinical use of 3D printing techniques would increase productivity and offer a more convenient method of fabricating provisional restorations. For a wider application of the 3D printing technology in dental care, additional studies are required to examine flexural, compressive, tensile, shear, and fatigue strength along with solubility and permeability. These physical properties of the 3D printed resin materials with respect to many factors should be studied in the future.

## 5. Conclusions

The use of resin materials in the 3D printing manufacturing for making a dental restoration is worthy of study in a novel way. The purpose of this study was to investigate the volume loss and the maximal depth loss of wear of the 3D printed resin material compared with the milled and the conventionally fabricated resin materials opposing zirconia and metal antagonists. Within the limits of this in vitro study, the wear resistance of the 3D printed resin material was in a range comparable to the milled or the conventionally fabricated resin materials. The 3D printing manufacturing appeared to be suitable for dental restorations.

## Figures and Tables

**Table 1:** Resin materials investigated in this study *.

Product	Manufacturer	Composition	Flexural Strength	Flexural Modulus	Batch Number
C&B	NextDent	Poly Methyl Methacrylate	80 MPa	2000 MPa	XM284N01
Vipi Block^®^ PMMA Monocolor	VIPI	Poly Methyl Methacrylate	100 MPa	2200 MPa	0000067727
Jet™	Lang Dental Mfg. Co., Inc.	Poly Methyl Methacrylate	68.3 MPa	1698 MPa	1430-14EP (Powder)1304-14AX (Liquid)

* written followed by the manufacturer’s information.

**Table 2:** Parameters of the chewing simulator.

Parameter	Characteristics
Weight per sample	5 kg
Cycle frequency	0.8 Hz
Vertical movement	5 mm
Horizontal movement	2 mm
Rising speed	55 mm/s
Descending speed	55 mm/s
Forward speed	55 mm/s
Backward speed	55 mm/s
Cold/hot bath temperature	5 °C/55 °C
Dwell time	60 s
